# Mitigating Postoperative Fistula Risks in Laparoscopic Pancreatic Enucleation: A Retrospective Study

**DOI:** 10.1245/s10434-024-16702-x

**Published:** 2024-12-22

**Authors:** Lin Li, Xuechuan Li, Ke Liu, Wenguang Wu, Maolan Li, Yingbin Liu

**Affiliations:** 1 Department of Biliary-Pancreatic Surgery, Renji Hospital Affiliated to Shanghai Jiao Tong University School of Medicine, Shanghai, China; 2 Department of General Surgery, Jiading Branch, Renji Hospital Affiliated to Shanghai Jiao Tong University School of Medicine, Shanghai, China; 3 Shanghai Key Laboratory of Systems Regulation and Clinical Translation for Cancer, Shanghai, China; 4 State Key Laboratory of Systems Medicine for Cancer, Shanghai Cancer Institute, Shanghai, China

**Keywords:** Pancreatic enucleation, Pancreatic fistula, Pancreatic duct stenting, ERCP

## Abstract

**Background:**

Pancreatic enucleation is a reliable surgical method for treating benign and borderline pancreatic tumors; however, the incidence of postoperative pancreatic fistula (POPF) is high, especially when the tumor is close to the main pancreatic duct. This study aimed to reduce the incidence of pancreatic fistula by preoperative placement of pancreatic stents and to summarize our center's experience with this procedure.

**Methods:**

From June 2020 to July 2023, patients diagnosed with benign or borderline pancreatic tumors at Renji Hospital were included. The pancreatic duct stent is placed through endoscopic retrograde cholangiopancreatography on the day of surgery or 1 day before surgery. The effectiveness of preoperative pancreatic stent placement in reducing pancreatic fistula was investigated by comparing the incidence of perioperative and postoperative complications.

**Results:**

Overall, 63 patients were included in the study, 41 of whom had preoperative pancreatic stents. Multivariate logistic regression showed that tumors located in proximity to the main pancreatic duct (≤ 2 mm) (odds ratio [OR] 5.58, *p* = 0.020) is an independent risk factor for pancreatic fistula, while preoperative stent placement (OR 0.23, *p* = 0.021) significantly reduces the occurrence of pancreatic fistula. There was no difference in the incidence of grade Ш–IV complications (*p* = 0.33) and postoperative pancreatitis (*p* > 0.99) between groups with or without pancreatic stent.

**Conclusion:**

Preoperative placement of pancreatic stents is associated with a lower incidence of pancreatic fistula, especially in patients with tumors adjacent to the main pancreatic duct. Moreover, preoperative pancreatic stents do not increase the incidence of postoperative pancreatitis or grade Ш–IV complications.

**Supplementary Information:**

The online version contains supplementary material available at 10.1245/s10434-024-16702-x.

With the rapid development of diagnostic imaging technology and the popularization of general health examinations, the detection rate of benign and borderline pancreatic tumors, including intraductal papillary mucinous neoplasm (IPMN), mucinous cystic neoplasm (MCN), serous cystic neoplasm (SCN), solid pseudopapillary tumor (SPT), and pancreatic neuroendocrine neoplasm (pNEN), has been increasing annually. Currently, pancreatic enucleation has been confirmed as a feasible surgical method for benign and borderline pancreatic tumors.^1,2^ This procedure maximizes the preservation of the endocrine and exocrine functions of the pancreas. Its characteristics of minimal tissue removal, rapid recovery, and preservation of gastrointestinal tract integrity can reduce the occurrence of postoperative complications and has been applied in multiple centers. However, the high postoperative pancreatic fistula (POPF) rate, varying from 9 to 69%,^3–8^ remains a major concern and the proximity of the lesion to the main pancreatic duct has been proven to be a risk factor.^3,9^ Various surgical improvements to reduce the incidence of pancreatic fistula are still under exploration.

Acute pancreatitis is another common complication following pancreatic resection and endoscopic retrograde cholangiopancreatography (ERCP) and is associated with various risk factors, including Sphincter of Oddi dysfunction (SOD), female sex, history of pancreatitis, hard pancreatic texture, and distance to the main pancreatic duct.^10,11^ Acute pancreatitis often coincides with pancreatic duct hypertension and obstruction, thereby increasing the risk of pancreatic fistula;^12,13^ however, postoperative pancreatitis after pancreatic nucleation has scarcely been investigated and its relationship with pancreatic fistula requires further exploration.

This study aimed to compare the incidence of POPF, pancreatitis, and other complications between groups with or without preoperative pancreatic stent, thereby exploring the impact of preoperative pancreatic stent placement on reducing pancreatic fistula.

## Methods

### Patient and Data Collection

From June 2020 to July 2023, patients with benign and borderline pancreatic tumors diagnosed at Renji Hospital affiliated to Shanghai Jiao Tong University School of Medicine were reviewed. All patients underwent pancreatic enhanced computed tomography (CT) and enhanced magnetic resonance imaging (MRI). If imaging suggested the presence of lesions with a maximum diameter >3 cm, mural nodules >5 mm, thickened or enhanced cyst walls, main pancreatic duct dilation >5 mm, lymphadenopathy, elevated carbohydrate antigen (CA) 19-9 levels, or lesion enlargement during follow-up, endoscopic ultrasound (EUS) examination was recommended. For surgical indications, patients with imaging-diagnosed pNEN (insulinomas and non-functional pNEN), MCN, PCN, or SCN with high-risk factors were advised for surgical intervention. In patients diagnosed with branch-duct IPMN (BD-IPMN) and presenting with risk factors as described above, surgery was also recommended. In cases where main-duct IPMN, mixed-type IPMN, or pNEN (excluding insulinomas) was considered, standard pancreatic resection was advised.

Patients who underwent pancreatic enucleation were included based on the following criteria: (1) The tumor was located <4 mm from the main pancreatic duct; (2) dilation of the main pancreatic duct; and (3) the tumor had no clear boundaries and preoperative assessment indicated substantial loss of the pancreatic parenchyma was expected during surgery. Patients meeting any of the above inclusion criteria underwent preoperative consultation for pancreatic duct stent placement. Clinicopathological data, including basic information, preoperative tests, imaging data, operative records, postoperative complications, pathology, and follow-up information, were systematically extracted. Two pancreatic surgeons reviewed the preoperative MRI and CT scans to measure the distance between the lesion and the main pancreatic duct. For patients who underwent EUS, the distance between the tumor and the main pancreatic duct was determined by the EUS report. The study was approved by the Ethics Review Committee of Renji Hospital affiliated to Shanghai Jiao Tong University School of Medicine (KY-2021-231).

### Pancreatic Stent Placement

Of the 63 included patients, 45 agreed to the preoperative placement of a pancreatic duct stent, and, ultimately, 41 successfully underwent stent implantation through ERCP. The stents used were 5 Fr or 7 Fr in diameter, with lengths varying from 7 to 9 cm, depending on tumor location. In cases where the lesion was close to the common bile duct and the boundary with the surrounding tissue was unclear, both pancreatic and biliary stents were placed. The stents were removed via ERCP 2–4 months postoperatively.

### Intraoperative and Postoperative Management

All patients underwent laparoscopic surgery. Intraoperative ultrasound was employed as necessary, based on the proximity of the lesion to the main pancreatic duct and bile duct. The surgical field was carefully inspected after excision of the lesion. Main pancreatic duct pinhole-like defects were directly sutured with 6-0 PDS polydioxanone synthetic absorbable sutures, and lateral continuous defects were sutured horizontally or vertically with 6-0 PDS or barbed sutures. For pancreatic head lesions close to the common bile duct, the position of the biliary stent was confirmed by compression, and the surrounding biliary tissue was carefully dissected while protecting the pancreatoduodenal arcade to ensure adequate blood supply to the duodenum and common bile duct. Intraoperative frozen sectioning was performed, and after pathology confirmation, careful hemostasis and clear drainage of the surgical area were ensured.

Blood amylase and drain fluid amylase levels were measured on postoperative days (PODs) 1 and 3. Patients started drinking water on day 1 post-surgery and progressed to a liquid diet by day 3. An abdominal CT scan was scheduled between days 3 and 5 post-surgery. All patients were treated with somatostatin for 3 days postoperatively, with the duration extended if a pancreatic fistula occurred. Food intake was delayed in cases of complications such as delayed gastric emptying, abdominal infection, or postoperative hemorrhage.

POPF is defined according to the International Study Group for Pancreatic Surgery (ISGPS) definition,^14^ while post-pancreatectomy acute pancreatitis (PPAP) is defined by postoperative hyperamylasemia (POH), which involves elevated serum amylase levels exceeding the normal upper limit on PODs 1–2, along with radiological evidence of pancreatitis on a postoperative CT scan.^11^ The Clavien–Dindo classification system evaluates the severity of postoperative complications. Major complications were categorized as grade III–IV and were identified within 60 days following surgery.^15^ Post-pancreatectomy hemorrhage (PPH) was defined according to the ISGPS definition.^16^ Exocrine pancreatic insufficiency was indicated by the need for oral pancreatic enzyme supplements at the last follow-up visit. All patients were regularly followed-up at the outpatient clinic after discharge.

### Data Analysis

Continuous variables are expressed as mean ± standard deviation or median and interquartile range, and categorical variables are presented as frequencies and percentages. Pearson’s Chi-square test and Fisher's exact test were used to compare categorical variables between groups. The Shapiro–Wilk test was used to check for normal distribution. For numerical variables with a non-normal distribution, the Wilcoxon rank-sum test was applied, whereas the t-test was used for those with a normal distribution. In logistic regression, variables with a *p*-value of <0.10 were included in the multivariate regression. Statistical significance was set at *p* < 0.05. All data analyses were performed using R version 4.3.2 (The R Foundation for Statistical Computing, Vienna, Austria).

## Results

### General Information

**Fig. 1:**
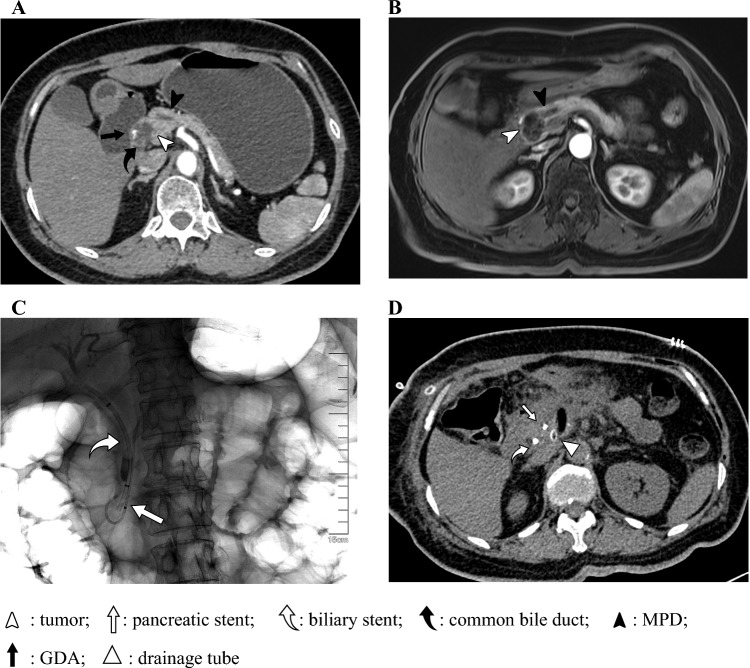
The patient had a 27 mm cystic lesion in the head of the pancreas. (**a**) Preoperative enhanced CT scan showed the tumor was in very close proximity to the GDA and the common bile duct. (**b**) Preoperative MRCP indicated mild dilation of the MPD, with the tumor potentially communicating with it. (**c**) Abdominal x-ray after biliary and pancreatic stent placement. (**d**) CT scan on POD 3 showed minimal intraperitoneal effusion. The blood amylase level was 87 U/L on POD 1, and postoperative pathology indicated a non-invasive IPMN with focal mild atypical epithelial hyperplasia. *GDA* gastroduodenal artery, *MPD* main pancreatic duct, *CT* computed tomography, *MRCP* magnetic resonance cholangiopancreatography, *POD* postoperative day, *IPMN* intraductal papillary mucinous neoplasm

**Fig. 2:**
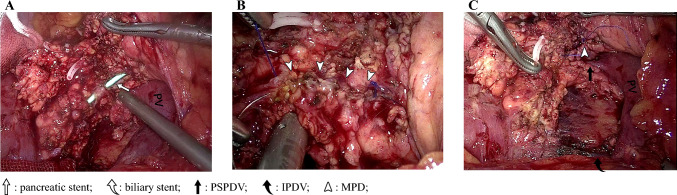
Intraoperative images. (**a**) Intraoperative continuous defects in the MPD. (**b**) Continuous suturing of the MPD with barbed sutures. (**c**) Hemostasis of the operative field. *MPD* main pancreatic duct

As shown in Table 1 and Supplementary Table 1, this study encompassed 63 individuals who underwent enucleation, including 26 males and 37 females. The median patient age was 52 years (45–57 years). Preoperative radiological assessments revealed that the lesion was ≤2 mm from the main pancreatic duct in 38 patients (60.3%) and >2 mm away in 25 patients (39.7%). All patients were informed of the preoperative pancreatic stents; 45 patients consented to preoperative stent placement, of whom 4 encountered insertion failures and 18 opted against the preoperative procedure. Among these patients, 8 received both biliary and pancreatic stent placements, while 33 patients had only pancreatic stents placed. Thirty-two patients had their pancreatic stents placed on the same day as surgery, others had their stents placed the day before surgery, and one patient had a stent placed 2 months prior to surgery due to pancreatitis. No recurrence or mortality was observed during follow-up. Graphics of typical patients are shown in Figs. 1 and 2 as well as electronic supplementary material (ESM) Fig. 1.

**Table 1:** Clinicopathological characteristics between groups with or without a pancreatic stent

Characteristic^a^	Overall [*n* = 63]	Unstented [*n* = 22]	Stented [*n* = 41]	*p* Value
Sex				0.12
Male	26 (39.7)	12 (54.5)	14 (34.1)	
Female	37 (60.3)	10 (45.5)	27 (65.9)	
Age, years (median (IQR)]	52 [45, 57]	56 [46, 57]	51 [44, 55]	0.34
BMI (mean ± SD)	24.13 ± 2.45	24.57 ± 2.57	23.89 ± 2.38	0.30
Diabetes	8 (12.7)	5 (22.7)	3 (7.3)	0.11
Smoker	6 (9.5)	4 (18.2)	2 (4.9)	0.17
ASA score				0.17
1	5 (7.9)	2 (9.1)	3 (7.3)	
2	54 (85.7)	17 (77.3)	37 (90.2)	
3	4 (6.3)	3 (13.6)	1 (2.4)	
Tumor location				0.78
Head and uncinate	33 (52.4)	11 (50.0)	22 (53.7)	
Body and tail	30 (47.6)	11 (50.0)	19 (46.3)	
Distance to MPD, mm				0.69
≤2	38 (60.3)	14 (63.6)	24 (58.5)	
>2	25 (39.7)	8 (36.4)	17 (41.5)	
Dilated MPD	27 (42.9)	7 (31.8)	20 (48.8)	0.19
Timing of ERCP				–
Same day as surgery	–	–	32 (78.0)	
Day before surgery	–	–	9 (22.0)	
Catheter				<0.001
None	22 (34.9)	22 (100)	0 (0)	
5Fr*7 cm	9 (14.3)	0 (0)	9 (21.9)	
5Fr*8 cm	26 (41.3)	0 (0)	26 (63.4)	
5Fr*9 cm	5 (7.9)	0 (0)	5(12.2)	
7Fr*7 cm	1 (1.6)	0 (0)	1 (2.4)	
Operation				0.68
EN	46 (73.0)	17 (77.3)	29 (70.7)	
EN + PDR	10 (15.9)	2 (9.1)	8 (19.5)	
EN + LC	7 (11.1)	3 (13.6)	4 (9.7)	
Intraoperative US				0.26
No	29 (46.0)	8 (36.4)	21 (51.2)	
Yes	34 (54.0)	14 (63.6)	20 (48.8)	
Surgery duration (median [IQR])	2.33 [1.75, 3.00]	3.00 [2.08, 3.19]	2.25 [1.66, 3.00]	0.046
Blood loss (median [IQR])	50 [10, 125]	62.5 [10, 120]	50 [10, 125]	0.68
Days to drain removal (median [IQR])	14 [10, 21]	18 [14, 24]	13 [8, 18]	0.016
Tumor size (median [IQR])	18 [14, 24]	18 [13, 26]	18 [14, 22]	0.83
Pathology				0.073
SCN^b^	10 (15.9)	7 (31.8)	3 (7.3)	
pNET G1	23 (36.5)	5 (22.7)	18 (43.9)	
pNET G2	5 (7.9)	1 (4.5)	4 (9.8)	
IPMN	16 (25.4)	6 (27.3)	10 (24.4)	
SPT	8 (12.7)	2 (9.1)	6 (14.6)	
Schwannoma	1 (1.6)	1 (4.5)	0 (0)	
Clavien–Dindo complication				0.33
<3	58 (92.1)	19 (86.4)	39 (95.1)	
≥3	5 (7.9)	3 (13.6)	2 (4.9)	
New-onset Diabetes	1 (1.6)	0 (0)	1 (2.4)	>0.99
Exocrine dysfunction^c^	2 (3.2)	0 (0)	2 (4.9)	0.54
BMI change (median [IQR])	−0.80 [−1.52, 0.00]	−1.29 [−1.85, −0.78]	−0.30 [−1.17, 0.00]	0.016

Data are expressed as *n* (%) unless otherwise specified

^a^All patients were of Asian descent

^b^The indications for enucleation in 10 SCN patients were as follows: 3 patients presented with main pancreatic duct dilation, 1 patient developed obstructive jaundice, 1 patient experienced pancreatitis with abdominal pain, 2 patients exhibited an increase in tumor size during follow-up and expressed significant preference for surgical intervention, and in 3 patients, the imaging studies were inconclusive, with postoperative pathology confirming SCN

^c^Our institution currently lacks FE-1 testing, thus pancreatic exocrine function mainly relies on clinical symptoms and documentation of oral administration of a pancreatic enzyme tablet

*BMI* body mass index, *ASA* American Society of Anesthesiologists, *MPD* main pancreatic duct, *EN* enucleation, *PDR* pancreatic duct repair, *LC* laparoscopic cholecystectomy, *US* ultrasound, *SCN* serous cystic neoplasm, *pNET* pancreatic neuroendocrine tumor, *IPMN* intraductal papillary mucinous neoplasm, *SPT* solid pseudopapillary tumor, *ERCP* endoscopic retrograde cholangiopancreatography, *FE-1* fecal elastase-1, *IQR* interquartile range, *SD* standard deviation

### Risk Factors for Pancreatic Fistula

As shown in Table 2, univariate analysis revealed that distance from the main pancreatic duct (odds ratio [OR] 0.19, *p* = 0.016), pancreatic stent (OR 0.24, *p* = 0.014), and duration of surgery (OR 2.41, *p* = 0.029) were associated with POPF. In multivariate logistic regression, the distance from the main pancreatic duct (OR 0.18, *p* = 0.020) and pancreatic stent placement (OR 0.23, *p* = 0.021) remained independent risk factors.

**Table 2:** Results of univariate and multivariate logistic regression analysis

Variable	Univariate analysis			Multivariate analysis		
	OR	95% CI	*p*- Value	OR	95% CI	*p* Value
Sex						
Female vs. male	0.51	0.17–1.53	0.23			
Age, years						
>52 vs. ≤52	2.48	0.81–7.51	0.11			
BMI						
>23.9 vs. ≤23.9	1.37	0.46–4.07	0.56			
Diabetes						
Yes vs. no	2.67	0.59–12.0	0.20			
Smoker						
Yes vs. no	0.43	0.05–3.98	0.46			
ASA score						
3 vs. 1 or 2	0.75	0.07–7.80	0.82			
Tumor location						
Head and uncinate						
Body and tail	0.53	0.18–1.61	0.26			
Distance to MPD						
>2 vs. ≤2 mm	0.19	0.05–0.74	**0.016**	0.18	0.04–0.76	**0.020**
Dilated MPD						
>5 vs. ≤5 mm	1.98	0.64–6.15	0.24			
Timing of ERCP						
Before vs. same day	1.24	0.20–7.53	0.82			
Preoperation stent						
Yes vs. no	0.24	0.07–0.76	**0.014**	0.23	0.07–0.79	**0.021**
Operation						
EN						
EN + PDR	1.69	0.41–6.99	0.47			
EN + LC	1.02	0.18–5.91	0.98			
Surgery duration						
>2.33 vs. ≤2.33 h	2.41	1.09–5.32	**0.029**	1.70	0.50–5.79	0.39
Blood loss						
>50 vs. ≤50 mL	0.66	0.22–1.97	0.46			
Tumor size						
>18 vs. ≤18 mm	1.76	0.60–5.23	0.31			
Pathology	NA		0.99			

Statistically significant *p*-values are given in bold

*OR* odds ratio, *CI* confidence interval, *BMI* body mass index, *ASA* American Society of Anesthesiologists, *MPD* main pancreatic duct, *EN* enucleation, *PDR* pancreatic duct repair, *LC* laparoscopic cholecystectomy, *NA* not available, *ERCP* endoscopic retrograde cholangiopancreatography

### Postoperative Outcomes

**Fig. 3:**
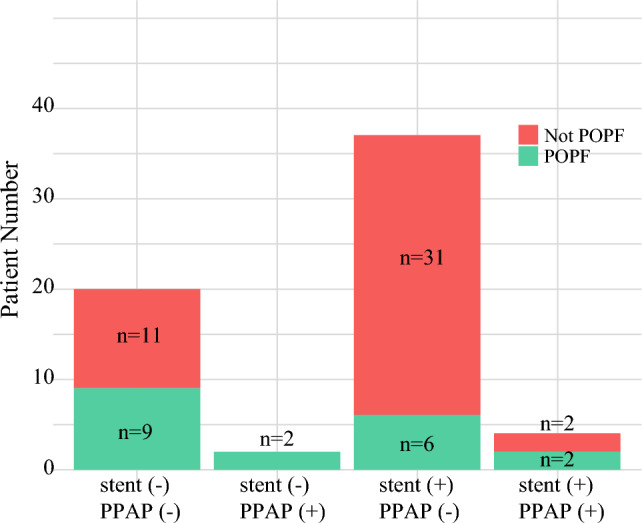
Relations between pancreatic fistula, preoperative stent placement, and PPAP. *PPAP* post-pancreatectomy acute pancreatitis, *POPF* postoperative pancreatic fistula

During hospitalization, there was no difference in grade III–IV complications (*p* = 0.33) or the incidence of postoperative pancreatitis between the stent and non-stent groups (*p* > 0.99). Nineteen (30.2%) individuals developed grade B or C pancreatic fistulas, 12 (19.0%) exhibited drainage durations exceeding 3 weeks postoperatively, 3 (4.7%) underwent percutaneous drainage, and 1 (1.5%) received a second open surgery due to postoperative hemorrhage (Table 3). Six patients were diagnosed with postoperative pancreatitis, of whom four experienced pancreatic leaks. Among the 57 patients not diagnosed with pancreatitis, 15 developed pancreatic leaks (*p* = 0.062). The relationship between stent placement, distance from the main pancreatic duct, PPAP, and pancreatic leaks is shown in Fig. 3. During the postoperative follow-up, one patient developed new-onset diabetes post-surgery, one diabetic patient achieved remission without requiring exogenous hypoglycemic drugs or insulin, and two (3.2%) patients still required oral pancreatic enzyme supplementation 6 months post-surgery.

**Table 3:** Complications between groups with or without pancreatic stents

	Unstented [*n* = 22]	Stented [*n *= 41]	*p* Value
Pancreatic fistula (B/C)	11	8	0.026
PPAP	2	4	>0.99
Pancreatic pseudocyst	1	0	0.35
Peritoneal effusion	0	2	0.54
Abdominal infection	0	1	>0.99
Postoperative hemorrhage	1	0	0.35

*PPAP* post-pancreatectomy acute pancreatitis

## Discussion

Benign and borderline pancreatic tumors typically occur at a younger age and have a favorable prognosis with a very low probability of tumor recurrence. The latest National Comprehensive Cancer Network (NCCN) guidelines recommend enucleation for exophytic insulinomas or benign tumors distant from the main pancreatic duct, whereas standard pancreatic resection is recommended for tumors close to the pancreatic duct.^17^ Traditional pancreatic surgery, which removes a significant amount of normal pancreatic tissue, can lead to endocrine and exocrine dysfunction, increasing the risk of postoperative diabetes and affecting the quality of life. Pancreatoenterostomy disrupts normal gastrointestinal integrity, leading to major complications such as fistula, abdominal bleeding, gastrointestinal dysfunction, and perioperative mortality. However, with the rapid advancement of minimally invasive surgical techniques and a deep understanding of the pancreatic anatomy, an increasing number of patients with benign and low-grade malignant pancreatic tumors are opting for pancreatic enucleation instead of conventional pancreatoduodenectomy or distal pancreatectomy. These patients have a higher risk of injury to the main pancreatic duct and subsequent development of pancreatic fistula, posing a serious challenge for pancreatic surgeons.

In 2012, Misawa et al.^18^ and Giuliani et al.^6^ reported the application of pancreatic enucleation with preoperative pancreatic stent placement in pancreatic neuroendocrine tumors, despite the small sample sizes. Xu et al.^9^ conducted a study of 18 patients with lesions close to the main pancreatic duct, in which pancreatic stents were placed to reduce the rate of clinically relevant POPF (CR-POPF). Multivariate analysis showed that pancreatic stent placement significantly reduced the incidence of pancreatic leaks (*p *= 0.028). The advantages of pancreatic duct stent placement during surgery include the following. First, its tactile feedback helps ascertain the position of the bile and pancreatic ducts and the pancreatic duct does not easily shift during tumor retraction due to the support of the stent, thus effectively preventing damage to the bile and pancreatic ducts. Second, the color contrast between the stent and pancreatic parenchyma aids in the immediate detection of pancreatic duct disruption. Third, the stent promotes drainage of bile and pancreatic fluids, reducing the intracavitary pressure of the bile and pancreas, thereby preventing postoperative pancreatitis and fistula. Finally, the stent effectively supports the bile and pancreatic ducts, preventing long-term complications such as postoperative collapse of these ducts and delayed pancreatic duct stricture.

As early as 1950, it was reported that pancreatic patients exhibited elevated serum amylase levels postoperatively, with a minority developing severe acute pancreatitis after surgery.^19^ The ISGPS established a consensus and grading for postoperative acute pancreatitis (PPAP) based on postoperative serum hyperamylasemia (POH) and radiologic findings,^11^ recognizing PPAP as an independent complication, which promotes the occurrence of pancreatic fistula.^20^ Recent studies have highlighted that pancreatic duct obstruction and hypertension, leading to impaired pancreatic fluid drainage, are significant mechanisms underlying postoperative pancreatitis, whereas the placement of pancreatic duct stents can reduce intraductal pressure and facilitate drainage, thereby lowering the incidence of postoperative pancreatitis without affecting postoperative recovery.^21–23^ Similarly, we observed a positive correlation between postoperative pancreatitis and pancreatic fistula (*p* = 0.062). Although this result did not reach the standard for statistical significance, it still indicates the necessity for timely clinical decision adjustments upon the emergence of hyperamylasemia. These adjustments include extending fasting periods, and using somatostatin analogs and antibiotics to manage postoperative pancreatitis, aiming to prevent and mitigate pancreatic fistula.

In patients whose tumors were located more than 4 mm from the main pancreatic duct and exhibited a complete capsule based on preoperative assessments, regular pancreatic nucleation was performed without preoperative stent placement (ESM Fig. 2 and ESM Fig. 3). For patients facing greater surgical complexity, our center proposes the following experience summaries. First, conduct a precise preoperative assessment combining CT, MRI, and EUS to determine the lesion type and the position of the main pancreatic duct and surrounding vessels, using intraoperative ultrasound flexibly to assess the main pancreatic duct. Second, protect the pancreatoduodenal arcade to ensure blood supply to the duodenum and common bile duct. When the lesion is closely related to the bile duct, a biliary stent plus pancreatic stent is inserted preoperatively, and the surrounding tissue around the common bile duct is dissected during surgery. Third, when the main pancreatic duct defect is unavoidable, suture the defect against the stent, extend the fasting period, and increase the duration of somatostatin use. Fourth, for patients with pancreatic fistula, removal of the drainage tube should be slow and staged to facilitate sinus tract closure.

There are several controversies regarding the use of stents. First, some surgeons choose to insert a pancreatic duct stent through the rupture into the main pancreatic duct, followed by suturing of the pancreatic parenchyma and embedding the main pancreatic duct.^24^ However, this is only suitable for patients with marked intraoperative main pancreatic duct damage, as it is less effective in preventing pinhole leaks. Second, common complications following ERCP include hemorrhage, perforation, and pancreatitis, with post-ERCP pancreatitis (PEP) being the most prevalent and clinically significant. Some scholars contend that the placement of a pancreatic stent might increase the incidence of cholangitis, pancreatic necrosis, and pancreatic cysts. Removing the stent may also increase patient discomfort and medical costs.^25,26^ On the other hand, in this study, stent placement did not increase postoperative complications, and in cases of long-term pancreatic fistula or pancreatic pseudocysts, the placement of a main pancreatic duct stent facilitates drainage as a therapeutic measure. Surgeons must balance the risks and benefits of this procedure based on their proficiency in performing ERCP. In our hospital, most patients underwent ERCP and pancreatic enucleation on the same day to mitigate the complications associated with pancreatic edema, which can increase surgical difficulty. Additionally, the routine pre-ERCP administration of rectal non-steroidal anti-inflammatory drugs (NSAIDs) was employed to decrease the likelihood of pancreatitis. Third, there is debate over the optimal management strategy for patients experiencing failure of pancreatic stent placement during ERCP. Some scholars believe that unsuccessful pancreatic stent placement independently elevates the risk for PEP, and suggest delaying any further invasive procedures until pancreatic enzymes have normalized, which might typically take up to 2 weeks. On the other hand, other studies suggest that performing an early cholecystectomy after ERCP is feasible and safe, even in cases of biliary pancreatitis.^27^ Our center's approach involves the following. First, highly experienced doctors generally complete the procedure within 10 min. Second, difficult cannulations are promptly abandoned to prevent repeated pancreatic duct manipulations and minimize complications related to ERCP. In this study, four patients experienced cannulation failures, yet underwent surgery on the same day as the ERCP without increased surgical difficulty. Postoperative outcomes included two cases of grade B pancreatic fistula and no pancreatitis. Management of such patients warrants further discussion. Third, there is currently no consensus regarding the timing of stent removal. Fourth, the use of stents should take into account the patient's economic situation and the healthcare burden.

This study had some limitations. First, its retrospective nature requires further validation. Second, the small number of patients in the non-stent placement group led to category imbalance and data sparsity, causing instability in the assessment. Third, in the non-stent placement group, four individuals experienced insertion failure, suggesting pancreatic duct stenosis or anatomic anomaly, which could increase the likelihood of pancreatic fistula in this group. Further randomized controlled trials are needed to validate its effectiveness, along with exploration of some controversies, such as the timing of stent removal, the economic burden brought by ERCP, and the management strategies for patients with failed stent placement.

## Conclusion

This study demonstrated that the distance to the pancreatic duct is an independent risk factor for pancreatic fistula, and preoperative placement of pancreatic duct stent is associated with a lower incidence of pancreatic fistula. Randomized trials of the significance of pancreatic stents are needed as more pancreatic nucleation is being performed at high-volume tertiary hospitals.

## Supplementary Information

Below is the link to the electronic supplementary material.

Supplementary file 1 (DOCX 1420 KB)
