# Supplementary material for: Mitigating Postoperative Fistula Risks in Laparoscopic Pancreatic Enucleation: A Retrospective Study
Source: Ann Surg Oncol. 2024 Dec 22;32(3):1887–95. doi: 10.1245/s10434-024-16702-x (PMC11811477; doi:10.1245/s10434-024-16702-x)
Supplement: Supplementary file 1 — (DOCX 1420 KB) [file 10434_2024_16702_MOESM1_ESM.docx]

**Supplementary Fig 1**


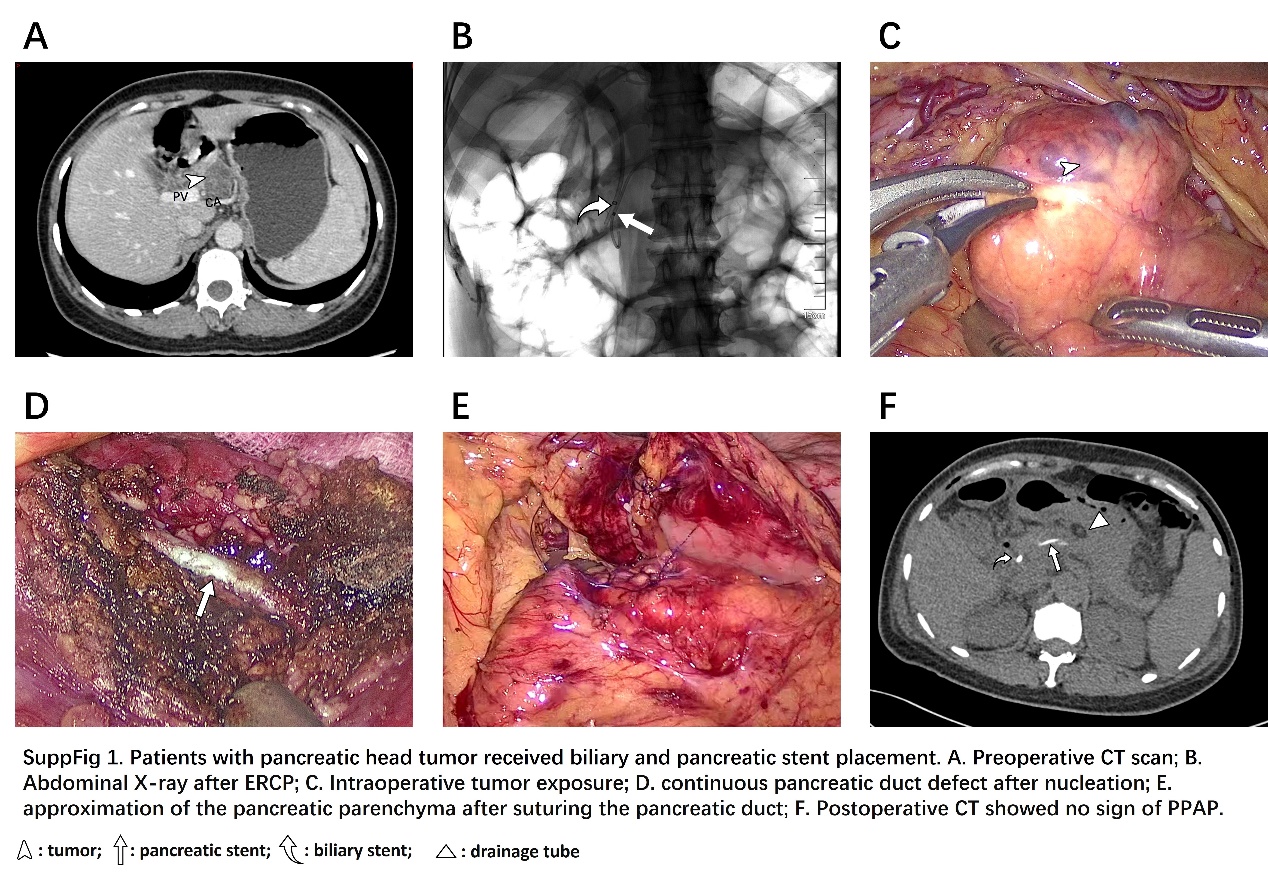


**Supplementary Fig 2**


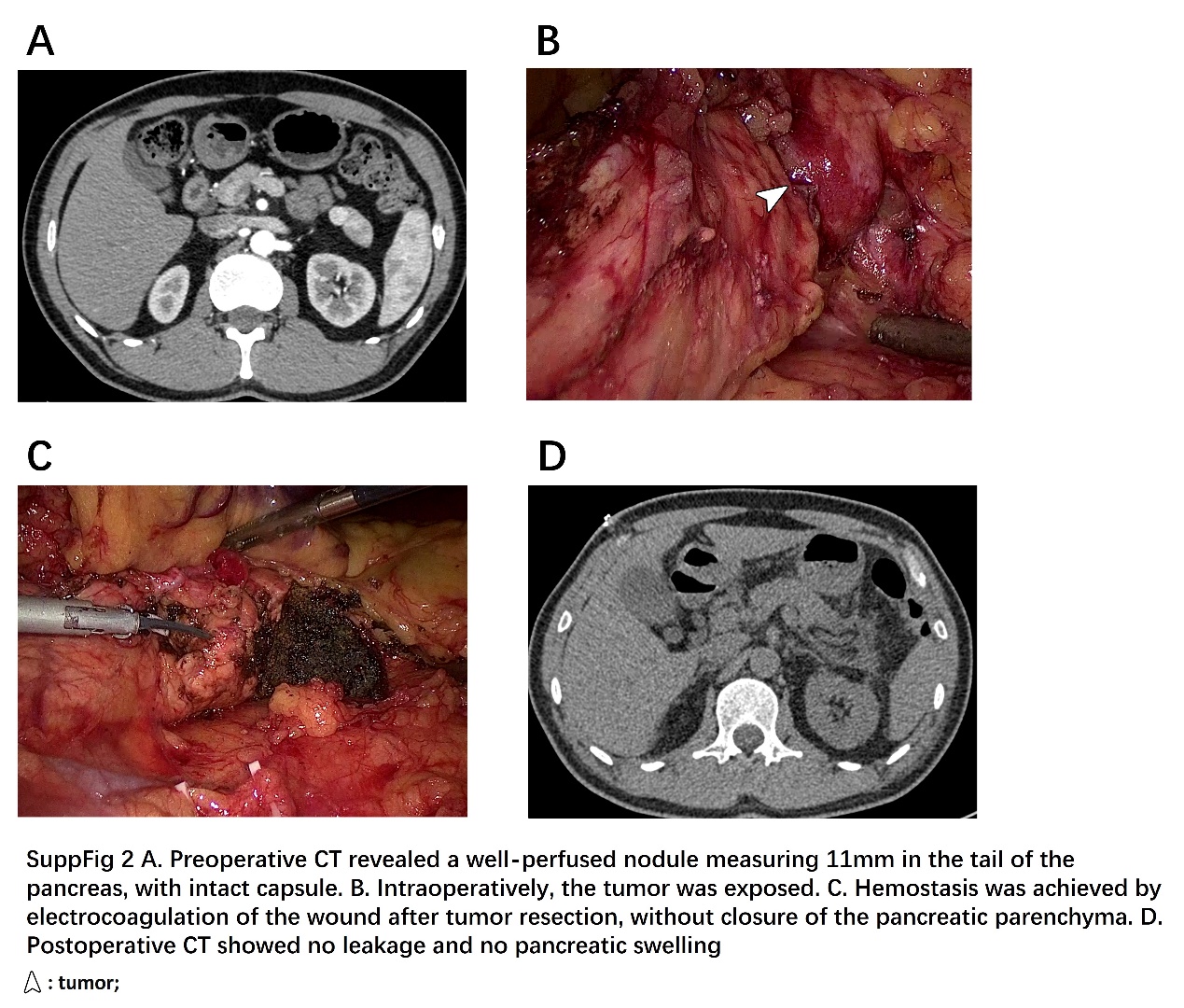


**Supplementary Fig 3**


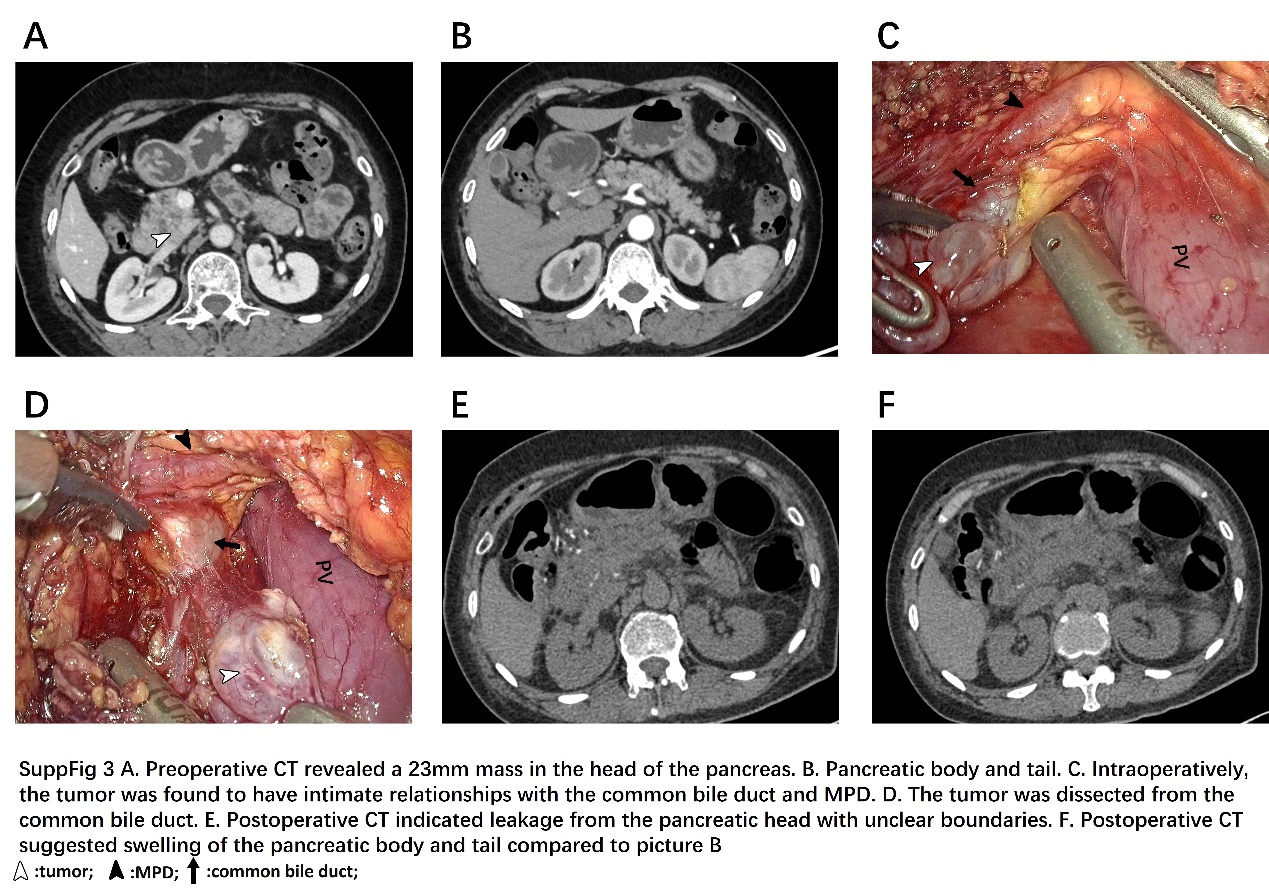


| **Supplementary Table 1. Comparison Between Groups With or Without Pancreatic Fistula** | | | | |
| --- | --- | --- | --- | --- |
| **Characteristic^1^** | **Overall (63)** | **POPF- (44)** | **POPF+ (19)** | **P value** |
| Sex |  |  |  | 0.23 |
| Male | 26 (41.2%) | 16 (36.4%) | 10 (52.6%) |  |
| Female | 37 (58.7%) | 28 (63.6%) | 9 (47.4%) |  |
| Age | 52 [45, 57] | 50 [44, 55] | 56 [47, 59] | 0.064 |
| BMI | 24.13 ±2.45 | 23.86±2.55 | 24.76 ±2.13 | 0.24 |
| Diabetes | 8 (12.7%) | 4 (9.1%) | 4 (21.1%) | 0.23 |
| Smoke | 6 (9.5%) | 5 (11.4%) | 1 (5.3%) | 0.66 |
| ASA |  |  |  | 0.30 |
| 1 | 5 (7.9%) | 2 (4.5%) | 3 (15.8%) |  |
| 2 | 54 (85.7%) | 39 (88.6%) | 15 (78.9%) |  |
| 3 | 4 (6.4%) | 3 (6.8%) | 1 (5.3%) |  |
| Tumor Location |  |  |  | 0.26 |
| Head and Uncinate | 33 (52.4%) | 21 (47.7%) | 12 (63.2%) |  |
| Body and Tail | 30 (47.6%) | 23 (52.3%) | 7 (36.8%) |  |
| Distance to MPD |  |  |  | **0.011** |
| ≤2mm | 38 (60.3%) | 22 (50.0%) | 16 (84.2%) |  |
| > 2mm | 25 (39.7%) | 22 (50.0%) | 3 (15.8%) |  |
| Dilated MPD | 27 (42.9%) | 21 (47.7%) | 6 (31.8%) | 0.23 |
| Timing of ERCP |  |  |  | **0.045** |
| Same day as surgery | 32 (50.8%) | 26 (59.1%) | 6 (31.6%) |  |
| Day before surgery | 9 (14.3%) | 7 (15.9%) | 2 (10.5%) |  |
| Pancreatic stent placement |  |  |  | **0.039** |
| None | 22(34.9%) | 11(25.0%) | 11(57.9%) |  |
| Pancreatic | 33(52.4%) | 27(61.4%) | 6(31.6%) |  |
| Pancreatic+biliary | 8(12.7%) | 6(13.6%) | 2(10.5%) |  |
| Catheter |  |  |  | 0.072 |
| None | 22 (34.9%) | 11 (25.0%) | 11 (57.9%) |  |
| 5Fr*7cm | 9 (14.3%) | 6 (13.6%) | 3 (15.8%) |  |
| 5Fr*8cm | 26 (41.3%) | 21 (47.7%) | 5 (26.3%) |  |
| 5Fr*9cm | 5 (7.9%) | 5 (11.4%) | 0 (0%) |  |
| 7Fr*7cm | 1 (1.6%) | 1 (2.3%) | 0 (0%) |  |
| Operation |  |  |  | 0.82 |
| EN | 46 (73.0%) | 33 (75.0%) | 13 (68.4%) |  |
| EN+PDR | 10(15.9%) | 6 (13.6%) | 4 (21.1%) |  |
| EN+LC | 7 (11.1%) | 5 (11.4%) | 2 (10.5%) |  |
| Intraoperative US |  |  |  | 0.039 |
| No | 29 (46.0%) | 24 (54.5%) | 5 (26.3%) |  |
| Yes | 34 (54.0%) | 20 (45.5%) | 14 (73.7%) |  |
| Surgery Duration | 2.33 [1.75, 3.00] | 2.29 [1.66, 3.00] | 3.00 [2.13, 3.25] | 0.018 |
| Blood Loss | 50 [10, 125] | 75 [10, 125] | 50 [10, 113] | 0.54 |
| Days to Drain Removal | 14 [10, 21] | 12 [8, 15] | 25 [22, 29] | **<0.001** |
| Tumor Size | 18 [14, 24] | 18 [13, 23] | 19 [16, 24] | 0.26 |
| Pathology |  |  |  | 0.083 |
| SCN^2^ | 10 (15.9%) | 8 (18.2%) | 2 (10.5%) |  |
| pNEN G1 | 23 (36.5%) | 16 (36.4%) | 7 (36.8%) |  |
| pNEN G2 | 5 (7.9%) | 3 (6.8%) | 2 (10.5%) |  |
| IPMN | 16 (25.4%) | 14 (31.8%) | 2 (10.5%) |  |
| SPT | 8 (12.7%) | 3 (6.8%) | 5 (26.3%) |  |
| Schwannoma | 1 (1.6%) | 0 (0%) | 1 (5.3%) |  |
| PPAP |  |  |  | 0.062 |
| No | 57(90.4%) | 42 (95.5%) | 15 (78.9%) |  |
| Yes | 6(9.6%) | 2 (4.5%) | 4 (21.1%) |  |
| Postoperative Bleeding | 1 (1.6%) | 0 (0%) | 1 (5.3%) | 0.30 |
| Clavien Dindo complication |  |  |  | 0.16 |
| <3 | 58 (92.1%) | 42 (95.5%) | 16 (84.2%) |  |
| ≧3 | 5 (7.9%) | 2 (4.5%) | 3 (15.8%) |  |
| New-onset Diabetes | 1 (1.6%) | 1 (2.3%) | 0 (0%) | >0.99 |
| Exocrine dysfunction^3^ | 2 (3.2%) | 1 (2.3%) | 1 (5.3%) | 0.52 |
| BMI Change | -0.80 [-1.52, 0.00] | -0.62 [-1.51, 0.00] | -0.14 [-1.52, -0.54] | 0.10 |
| BMI, Body mass index; ASA, American Society of Anesthesiologists score; MPD, Main pancreatic duct; EN, Enucleation; PDR, Pancreatic Duct Repair; LC, Laparoscopic Cholecystectomy; US, Ultrasound; SCN, Serous Cystic Neoplasm; pNEN, Pancreatic Neuroendocrine Neoplasm; IPMN, Intraductal Papillary Mucinous Neoplasm; SPT, Solid Pseudopapillary Tumor; PPAP, Post-pancreatectomy acute pancreatitis | | | | |
